# Predicting variant histology in bladder cancer: the role of multiparametric MRI and vesical imaging-reporting and data system (VI-RADS)

**DOI:** 10.1007/s00261-025-04852-9

**Published:** 2025-03-18

**Authors:** Serdar Aslan, Merve Nur Tasdemir, Ertugrul Cakir, Ural Oguz, Birgul Tok

**Affiliations:** 1 Department of Radiology, Faculty of Medicine, Giresun University, Giresun, Turkey; 2 Department of Urology, Faculty of Medicine, Giresun University, Giresun, Turkey; 3 Department of Pathology, Faculty of Medicine, Giresun University, Giresun, Turkey

**Keywords:** Bladder cancer, Variant histology, Magnetic resonance imaging, VI-RADS, Signal intensity heterogeneity, Necrosis

## Abstract

**Objectives:**

(1) To evaluate the diagnostic performance of the VI-RADS score in detecting muscle invasion in variant urothelial carcinomas (VUC). (2) To identify spesific MRI features that may serve as predicting for VUC.

**Methods:**

Two hundred four patients who underwent TUR-B and/or radical cystectomy and a bladder mpMRI scan within three months prior to the procedure were retrospectively enrolled. The tumors were divided into two histological cohorts: pure urothelial carcinoma (PUC) and VUC. Various MRI features, including largest tumor diameter, long-to-short axis ratio, morphology, heterogeneous signal intensity (SI), presence of necrosis, and normalized ADC (ADC_n_) value, were analyzed. The diagnostic performance of the VI-RADS score in predicting muscle invasion was calculated using a cut-off point of ≥ 4 in both cohorts. Univariate logistic regression were also performed to identify MRI features that predict VUC. Inter-reader agreement was assessed with the weighted kappa coefficient.

**Results:**

Our study identified several MRI features significantly associated with VUC, including heterogeneous SI on T2-weighted images (OR: 3.055; 95% CI: 1.312–7.112; *p* < 0.001), dynamic contrast enhancement images (OR: 2.935; 95% CI: 1.263–6.821; *p* < 0.001), and the presence of necrosis (OR: 3.575; 95% CI: 1.798–7.107; *p* < 0.001). Additionally, ADC_n_ values were significantly lower in the VUC cohort (*p* = 0.003). The VI-RADS score demonstrated high diagnostic performance across both VUC and PUC cohorts, with sensitivity ranging from 94.4 to 86.8% (reader 1) and 94.2–82.2% (reader 2), specificity ranging from 100 to 94.6% (reader 1) and 100–94% (reader 2), and accuracy ranging from 96 to 90.6% (reader 1) and 96–88.2% (reader 2). The area under the curve (AUC) ranged between 0.972 and 0.972 (reader 1) and 0.838–0.781 (reader 2). No significant differences in diagnostic performance were observed between readers or cohorts (*p* > 0.05), and inter-reader agreement for VI-RADS scores was excellent for both cohorts.

**Conclusion:**

VI-RADS score can be used with high performance to detect muscle invasion in VUC, regardless of reader experience. Additionally, specific MRI features such as heterogeneous SI, the presence of necrosis, and ADC_n_ values can serve as potential predictors of VUC.

**Graphical Abstract:**

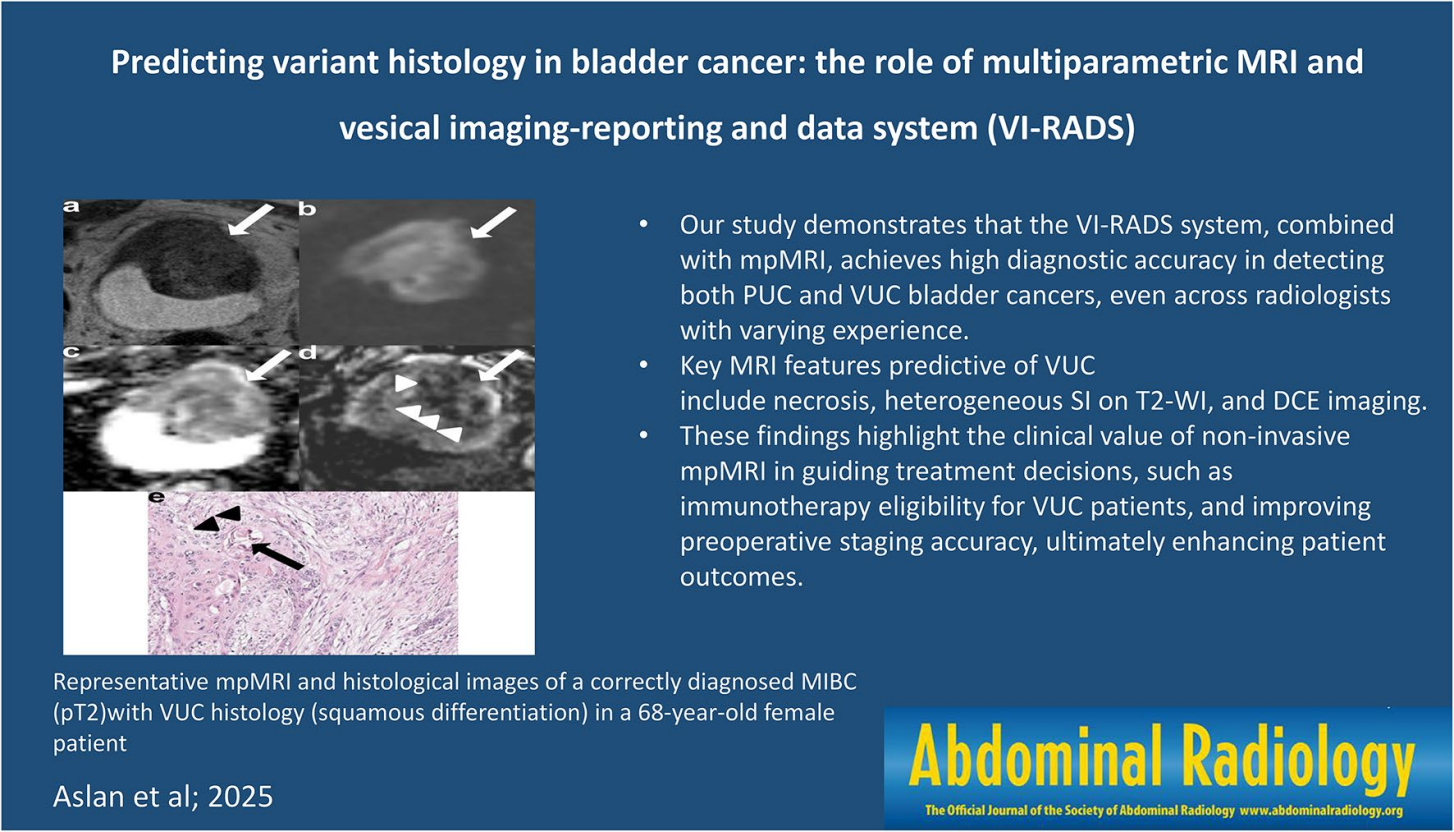

## Introduction

Bladder cancer (BC) ranks 10th malignancy worldwide, with approximately 550,000 new cases reported annually [1]. While most BC cases present as pure urothelial carcinoma (PUC) histology, 10–25% exhibit variant urothelial carcinoma (VUC) histology, including squamous, glandular, plasmacytoid, micropapillary, sarcomatoid, and nested subtypes [2, 3]. VUCs are associated with a worse prognosis than PUCs, often presenting as advanced, with a higher likelihood of being muscle-invasive at diagnosis [4, 5]. Additionally, muscle-invasive VUCs tend to show limited responsiveness to neoadjuvant chemotherapy (NAC) [6]. However, recent studies suggest that VUCs, particularly those with squamous differentiation, may respond favourably to immunotherapy due to increased programmed death-ligand 1 (PD-L1) expression [7]. Thus, distinguishing VUCs from PUCs is critical for guiding treatment.

Transurethral resection of the bladder (TUR-B) remains the gold standard for diagnosing and locally staging BC but notable limitations. Muscle invasion may be missed in approximately 25% of muscle-invasive BCs (MIBCs) due to superficial resection, with tumor stage often underestimated in large or multiple lesions [8, 9]. This issue is especially pronounced in VUCs, where the diagnostic performance of TUR-B is inconsistent. Mantica et al. reported only a 60% concordance rate between TUR-B and radical cystectomy (RC) specimens for VUC [10]. These challenges highlight the need for non-invasive imaging techniques to detect VUCs, preoperative staging, and refine treatments.

Multiparametric MRI (mpMRI), which combines T2-weighted imaging (T2-WI), diffusion-weighted imaging (DWI), and dynamic contrast-enhanced imaging (DCE), has become an accepted method for local staging of BC and for differentiating non-muscle-invasive BC (NMIBC) from MIBC [11–13]. The mpMRI-based Vesical Imaging-Reporting and Data System (VI-RADS) is a standardized reporting system used to assess muscle invasion in PUCs [11]. A meta-analysis by Jazayeri et al., which included 22 studies involving a total of 2,576 patients, demonstrated that VI-RADS has high diagnostic performance, with an area under the curve (AUC) of approximately 0.93 for diagnosing muscle invasion in PUCs [14]. While VI-RADS is effective in detecting muscle invasion in PUCs, its ability to accurately predict muscle invasion in VUCs is still under investigation [5, 15, 16]. Simplified visual and quantitative mpMRI markers that can help discriminate between PUCs and VUCs are also under investigation. The identification and utilization of these markers could increase the utility of the VI-RADS system integrated into mpMRI [17]. These markers include the apparent diffusion coefficient (ADC) value calculated from DWI, the largest tumor diameter, long-short axis ratio, tumor morphology, presence of necrosis and tumor signal intensity (SI) heterogeneity.

The aims of this study were (1) to assess the diagnostic performance of VI-RADS in detecting muscle invasion in VUCs and evaluate inter-reader agreement among individuals with varying levels of abdominal imaging experience and (2) to identify mpMRI features useful for predicting VUCs.

## Materials and methods

This single-centre retrospective study was approved by the institutional ethics committee (approval number: BAEK 2024/118). Due to the retrospective nature of the study, the requirement for informed consent was waived.

### Study population

**Fig. 1:**
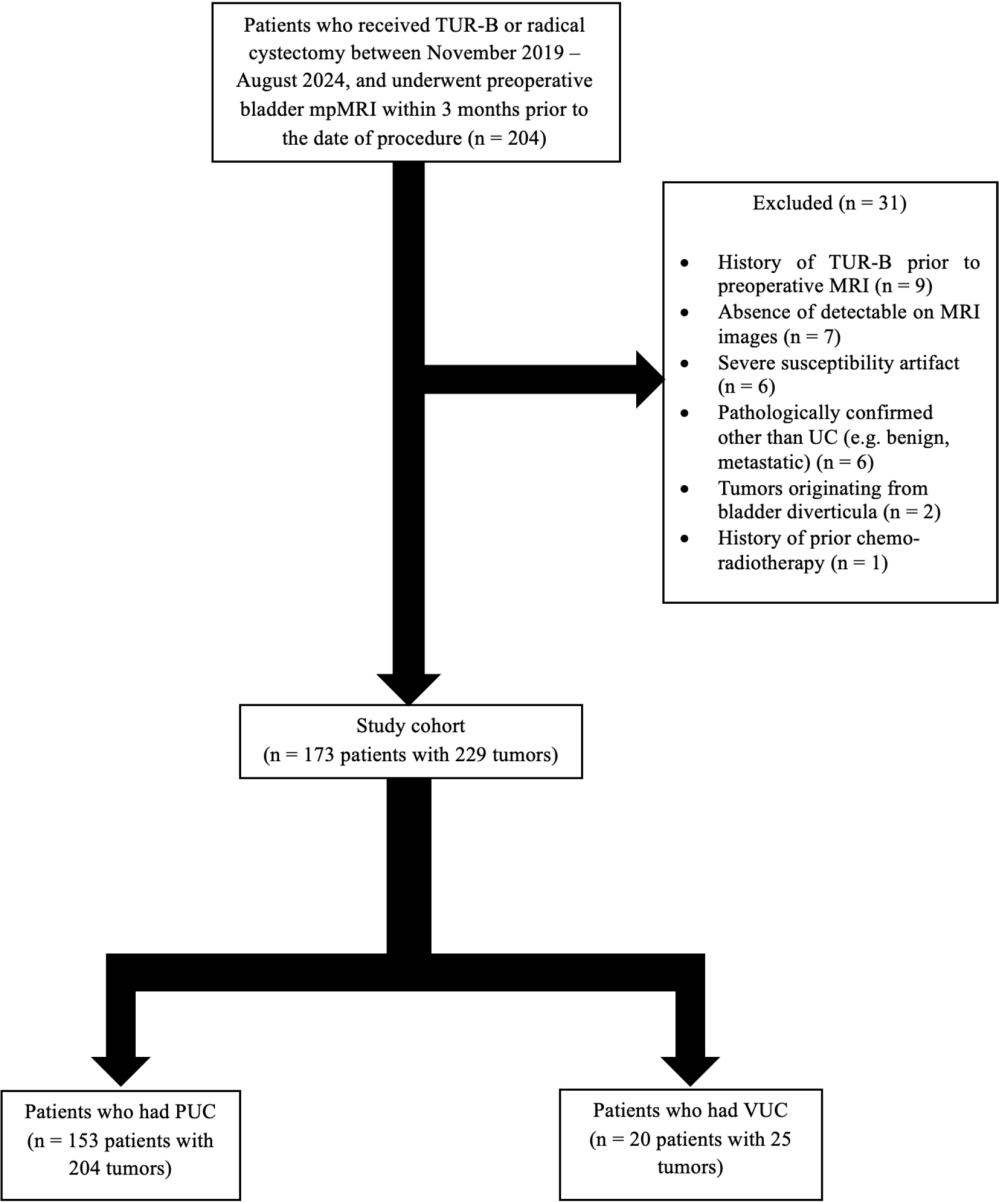
Flow-chart of patient selection

Between November 2019 and August 2024, 204 patients who underwent TUR-B and/or RC for BC with mpMRI performed within three months prior were identified. The inclusion criterion was the histopathologically confirmed diagnosis of bladder UC. Exclusion criteria included: (1) history of TUR-B prior to MRI (*n* = 9), (2) absence of detectable BC on MRI (*n* = 7), (3) severe susceptibility artifacts on MRI (*n* = 6), (4) pathologically confirmed diagnoses other than UC (e.g., benign, metastatic) (*n* = 6), (5) tumors originating from bladder diverticula (*n* = 2), and (6) history of prior chemo-radiotherapy (*n* = 1). For patients with multiple tumor foci, each tumor was evaluated individually. VI-RADS score was assigned to each. The final study population consisted of 173 patients with a total of 229 tumors. Figure 1 illustrates the patient selection process.

### Patient preparation for MRI

To adequate bladder distension, patients were instructed to urinate 1–2 h before the MRI and drink 500–1000 mL of water 30 min prior. Bladder filling was assessed using ultrasonography. Patients without contraindications were administered 20 mg of scopolamine butylbromide (Buscopan, Boehringer Ingelheim) intravenously to minimise artifacts.

### Image acquisition

All MRI examinations were conducted on a 1.5-T MRI system (Magnetom Aera, Siemens Medical Solutions, Erlangen, Germany). Images were obtained with the patient in the supine position using a 16-channel pelvic phased-array coil. The MRI protocol included the following sequences: unenhanced axial T1-WI, high-resolution three-plane T2-WI, axial DWI with b-values of 0, 800, and 1200 s/mm², and three-dimensional (3D) axial DCE T1-WI, including subtraction images. Gadopentetate dimeglumine (Gadovist; Bayer Healthcare, Berlin, Germany) was administered intravenously at a dose of 0.1 mL/kg at a rate of 2 mL/s, followed by a 20 mL saline flush. DCE images were acquired in 18 post-contrast phases, with each phase delayed by 10 s. Table 1 outlines the technical parameters of the MRI sequences.

**Table 1:** Technical details of mpMRI scanning parameters

	T2-WI	T1-WI	DWI	DCE
Imaging Planes Fat Saturation	Axial, coronal, sagittal -	Axial -	Axial -	Axial Fat-sat
Sequence	TSE	GRE	EPI	3D GRE
TE (ms)	100	8	90	3.9
TR (ms)	4500	600	4000	1.8
Flip Angle (degree)	90	90	90	12
NEX	2	1	12	1
FOV (cm)	18	30	21	30
Slice Thickness	3	3	3	3
Matrix size	256 × 164	376 × 325	108 × 85	152 × 150
b value (s/mm^2^)	-	-	0, 800, 1200	-
Temporal resolution (s)	-	-	-	10
Number of acquisitions	-	-	-	18

DWI sequences included ADC map calculation

mp-MRI, multiparametric magnetic resonance imaging; T2-WI, T2-weighted images; DWI, diffusion-weighted images; DCE, dynamic contrast enhancement; Fat-sat, fat saturated; TE, time to echo; TR, time to repetation; NEX, number of excitation; FOV, field of view

### Image analysis

All anonymised MR images were uploaded to a Picture Archiving and Communication System. The images were independently evaluated by a board-certified urogenital radiologist with 12 years of experience (reader 1) and a radiology intern with 3 years of experience (reader 2), whom were blinded to clinical and histopathological data. The largest tumor diameter and long-to-short axis ratio were recorded. The long-to-short axis ratio was calculated by dividing the length of the long axis by the length of the short axis perpendicular to it. Tumor morphology was characterised as papillary; broad-based, papillary; stalked, or flat in accordance with the VI-RADS v2018 guidelines [11]. ADC values of the tumors were calculated from DWI (b = 1200 s/mm²). The two 10mm^2^ ROIs were placed tumors avoiding necrosis and tumor stalk. The average ADC (ADC_tumor_) was calculated. To calculate the normalised ADC (ADC_n_), urine in the bladder lumen was used. ADC_urine_ obtained by placing the ROI in the centre of the bladder lumen. The ADC_n_ value was calculated using the formula ADC_tumor_/ADC_urine_. The presence of necrosis in the tumor was classified as absent (< 5%) or present (≥ 5%) based on the proportion of high SI on ADC map and the extent of contrast enhancement on DCE images. Tumor SI heterogeneity was defined as any focal tumor area exhibiting inhomogeneous SI on T2-WI, DWI, or DCE sequences. All tumors were scored in accordance with VI-RADS on T2-WI, DWI, and DCE images separately, and the final VI-RADS score was determined. Tumors with scores ≤ 3 were classified as NMIBC, while those with scores ≥ 4 were classified as MIBC [11–12].

### Standard of reference

RC (*n* = 55) was used as the reference standard to confirm the presence of muscle invasion. If RC was not performed or was performed with NAC, TUR-B (*n* = 118) served as the reference standard. To minimise discrepancies findings, a second TUR-B was performed in 90% of patients with high-risk NMIBC to remove suspicious residual tumor tissue and confirm disease stage, as recommended [5]. Specimens from RC and TUR-B were evaluated by a pathologist with 15 years of experience in uropathology, who was blinded to the MRI findings. Histopathological evaluation followed the 2016 World Health Organisation classification, with VUC defined as > 50% variant histology. [18].

### Statistical analysis

Statistical analyses were performed using SPSS software, v25 (IBM Corporation, Armonk, NY, USA). Continuous variables were expressed as mean ± standard deviation (SD) and analyzed using the Wilcoxon signed-rank test, while categorical variables were reported as numbers and percentages and analyzed using Fisher’s exact test. Diagnostic metrics for VI-RADS scores—including sensitivity, specificity, accuracy, and AUC—were calculated using a cutoff of ≥ 4 for muscle invasion for each reader and sequence. The Cochran-Armitage test was used to VI-RADS scores and muscle invasion status between PUC and VUC cohorts. Univariable logistic regression analysis was performed to identify independent predictors of VUC. Inter-reader agreement was using the weighted kappa (κ) coefficient, with κ values interpreted as follows: ≤0.40 (fair), 0.41–0.60 (moderate), 0.61–0.80 (good), and ≥ 0.81 (excellent). A p-value < 0.05 was considered statistically significant. All calculations were performed with a 95% confidence interval (CI).

## Results

### Demographic, radiological and pathological characteristics of patients

Table 2 summarizes the demographic, radiological, and pathological characteristics of the study population. Age and gender distribution did not differ significantly between the cohorts (*p* = 0.694 and 0.549, respectively). Muscle invasion and high tumor grade were significantly more common in the VUC cohort (*p* < 0.001). The largest tumor diameter was greater in the VUC cohort than in the PUC cohort for both readers (*p* < 0.001). However, there was no significant difference in the long-to-short axis ratio between the cohorts (*p* = 0.117 and 0.93 for readers 1 and 2, respectively). Tumor morphology did not significantly different between the cohorts (*p* = 0.546 and 0.478 for readers 1 and 2, respectively). Visual and quantitative MRI findings revealed that ADC_n_ values were significantly lower in the VUC cohort compared to the PUC cohort (*p* = 0.003 for both readers). Heterogeneous SI on T2-WI and DCE sequences, as well as tumor necrosis, were observed more frequently in the VUC cohort (*p* < 0.001; *p* < 0.001, 0.01, and < 0.001 for readers 1 and 2, respectively). However, no significant difference in heterogeneous SI on DWI was noted between the cohorts (*p* = 0.352 and 0.371 for readers 1 and 2, respectively).

**Table 2:** Demographic, radiological, and pathological characteristics of the patients

	PUC (*n* = 204)	VUC (*n* = 25)	*p* value
***Demographic Findings***			
**Sex, ** ***n*** ** (%)**			0.549
Male	132 (84.1)	11 (78.6)	
Female	27 (16.9)	3 (21.4)	
**Age (mean ± (SD))**	72.2 ± 10.4	73.1 ± 14.2	0.694
***Radiologic Findings***			
**Largest tumor diameter (mean ± [SD])** Reader 1 Reader 2	24.4 ± 16.9	43.8 ± 23.5	**< 0.001**
	25.3 ± 18.2	46.9 ± 25	**< 0.001**
**Long-to-short axis ratio (mean ± [SD])** Reader 1	1.78 ± 0.84	2.06 ± 0.87	0.117
Reader 2	1.91 ± 1.07	1.93 ± 0.88	0.93
**Tumor morphology**,** n (%)**			0.546
Papillary; broad-based	113 (55.4)	13 (52)	
Papillary; stalked	49 (24)	7 (28)	
Flat	42 (20.6)	5 (20)	
**ADC**_**n**_ **value (s/mm²)** Reader 1	0.43 ± 0.11	0.36 ± 0.81	**0.003**
Reader 2	0.42 ± 0.14	0.33 ± 0.1	**0.003**
**Heterogeneous SI on T2-WI**,** n (%)** Reader 1	37 (18.1)	16 (64)	**< 0.001**
Reader 2	43 (21)	14 (56)	**< 0.001**
**Heterogeneous SI on DWI**,** n (%)** Reader 1	98 (48)	13 (52)	0.371
Reader 2	102 (50)	12 (48)	0.352
**Heterogeneous SI on DCE**,** n (%)** Reader 1	30 (14.7)	16 (64)	**< 0.001**
Reader 2	55 (27)	13 (51)	**< 0.001**
**Presence of necrosis**,** n (%)** Reader 1	25 (12.3)	14 (56)	**< 0.001**
Reader 2	41 (20.1)	14 (56)	**< 0.001**
***Pathologic Findings***			
**Muscle invasion status**,** n (%)**			**< 0.001**
Muscle invasion	24 (11.7)	16 (36)	
No muscle invasion	180 (88.3)	9 (64)	
**T stage**,** n (%)**			**< 0.001**
Ta	97 (47.5)	1 (4)	
T1	83 (40.7)	8 (32)	
T2	21 (10.3)	6 (24)	
T3	2 (1)	8 (32)	
T4	1 (0.5)	2 (8)	
**Tumor grade**,** n (%)**			**< 0.001**
High	112 (54.9)	18 (72)	
Low	92 (45.1)	7 (28)	
**Pathological subtypes**,** n (%)**			N/A
Pure urothelial carcinoma	204 (100)	0 (0)	
Variant urothelial carcinoma Squamous differentiation		25 (100) 13 (52)	
Glandular differentiation		3 (12)	
Micropapillary variant		3 (12)	
Sarcomatoid variant		2 (8)	
Nested variant		2 (8)	
Plasmacytoid variant		1 (4)	
Lipid cell variant		1 (4)	

Bold values are statistically significant

PUC, pure urothelial carcinoma; VUC, variant urothelial carcinoma; SI, signal intensity

### Independent predictor features in mpMRI for the VUC cohort

Table 3 presents the results of univariable logistic regression analysis for predicting VUC. Heterogeneous SI on T2-WI and DCE sequences, the presence of necrosis were significant independent predictors of VUC (*p* < 0.001 for both readers). Inter-reader agreement for heterogeneous SI on T2-WI, DCE, and the presence of tumor necrosis were good, with κ values of 0.741, 0.703, and 0.685, respectively.

**Table 3:** Univariable logistic regression analysis for predicting VUC

Variables	Reader 1 OR (%95 CI) *p* value		Reader 2 OR (%95 CI) *p* value	
Heterogeneous SI on T2-WI	3.055 (1.312–7.112)	**< 0.001**	8.024 (3.292–19.558)	**< 0.001**
Heterogeneous SI on DCE	2.935 (1.263–6.821)	**< 0.001**	10.311 (4.176–25.46)	**< 0.001**
Presence of necrosis	3.575 (1.798–7.107)	**< 0.001**	6.44 (3.186–13.02)	**< 0.001**

Bold values are statistically significant

CI, confidence interval; OR, odds ratio

### Diagnostic performance of VI-RADS score in predicting muscle invasion

The distribution of VI-RADS scores for both readers in the PUC and VUC cohorts is presented in Table 4. VI-RADS scores were significantly higher in the VUC cohort compared to the PUC cohort for both readers (*p* < 0.001).

**Table 4:** Distribution of VI-RADS scores for both readers in the PUC and VUC cohorts*

VI-RADS Score	Reader 1			Reader 2		
	PUC (*n*%)	VUC (*n*%)	*p* value	PUC (*n*%)	VUC (*n*%)	*p* value
1	30 (14.7)	0 (0)	**< 0.001**	26 (12.7)	0 (0)	**< 0.001**
2	114 (55.9)	7 (28)		112 (54.9)	7 (28)	
3	24 (11.8)	1 (4)		33 (16.2)	1 (4)	
4	16 (7.8)	4 (16)		15 (7.4)	5 (20)	
5	20 (9.8)	13 (52)		18 (8.8)	12 (48)	

Bold values are statistically significant

VI-RADS, Vesical Imaging-Reporting and Data System;

* The distribution of VI-RADS scores between the PUC and VUC cohorts was compared using the Cochran-Armitage test

**Fig. 2:**
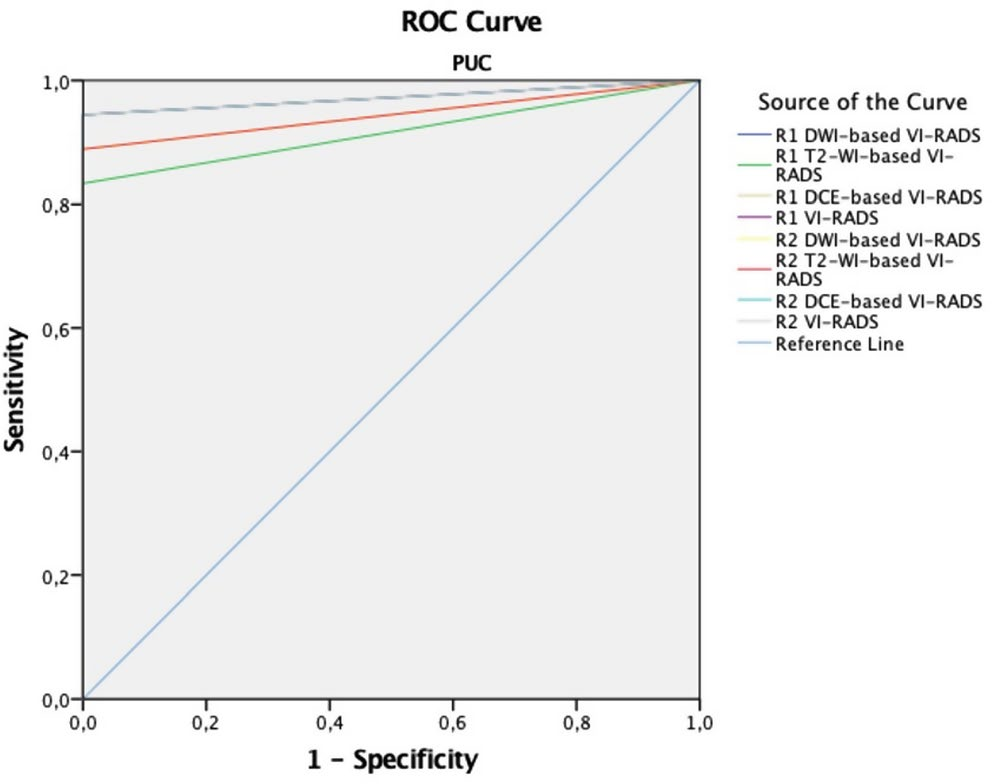
ROC curve for sequence-based and final VI-RADS score for MIBC detection in the PUC cohort

**Fig. 3:**
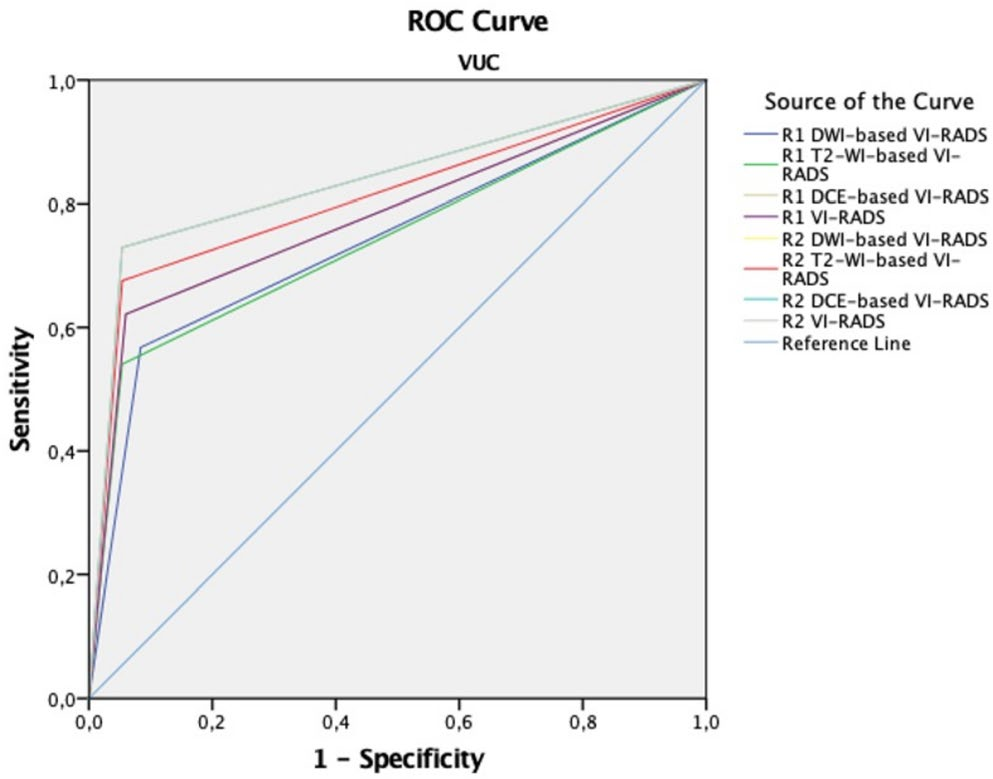
ROC curve for sequence-based and final VI-RADS score for MIBC detection in the VUC cohort

**Fig. 4:**
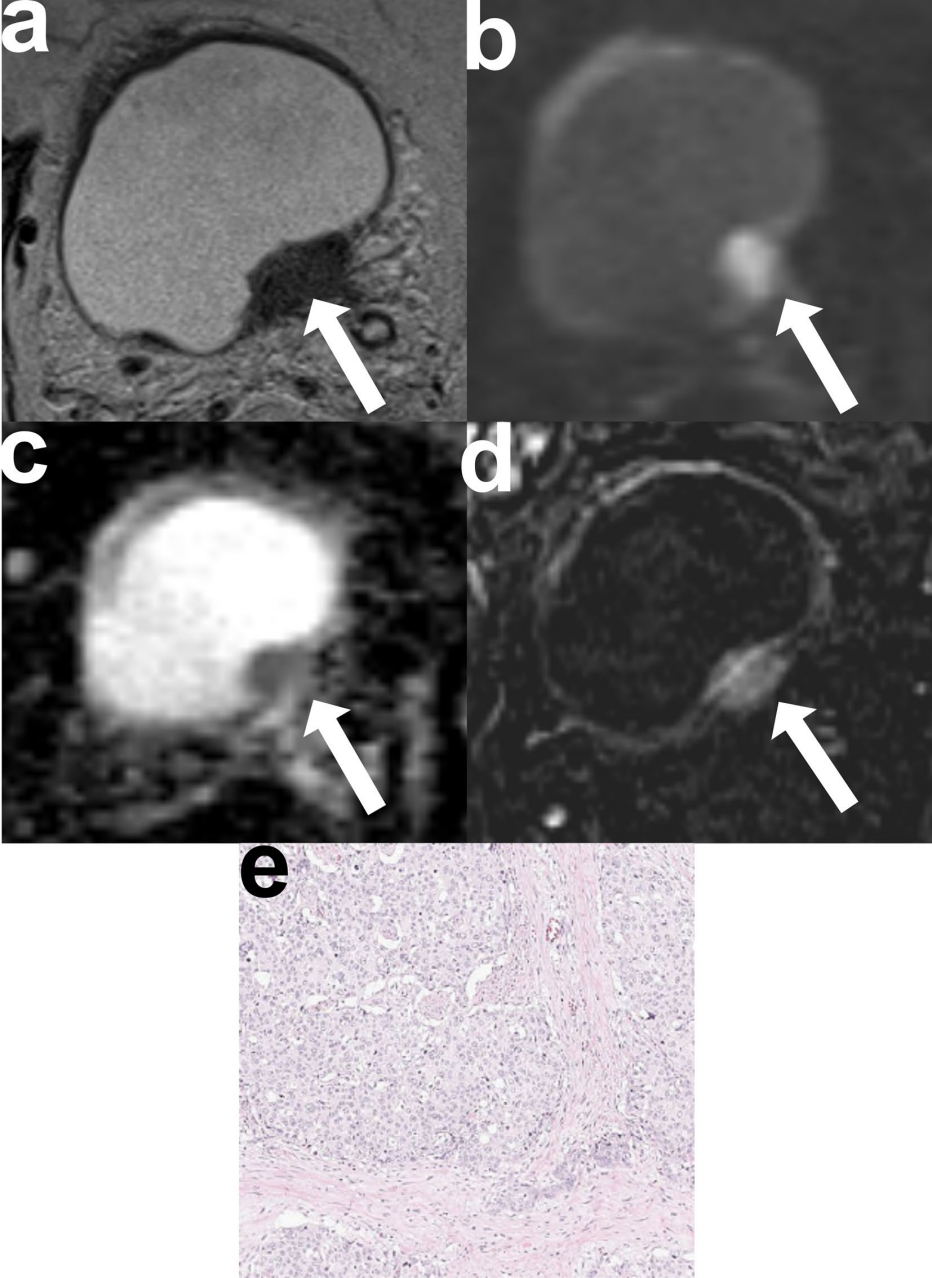
Representative mpMRI and histological images of a correctly diagnosed MIBC (pT2) with PUC histology in a 73-year-old male patient. **a**. Axial T2-WI sequence shows a broad-based; papillary tumor on the right posterolateral bladder wall with low SI. The tumor interrupts the low SI line of the muscular layer and extends into the perivesical fatty tissue (T2-WI-based score: 5 for both readers) (arrow). **(b)** DWI sequence clearly demonstrates a tumor with high SI that disrupts the low SI line of the muscular layer and extends into the perivesical fatty tissue (DWI-based score: 5 for both readers) (arrow). **(c)** ADC maps reveal the tumor’s low SI (arrow). **(d)** DCE sequence shows homogenous and early enhancement of the tumor with disruption of the muscular layer’s low SI, indicating tumor infiltration (arrow). The tumor also extends into the perivesical fatty tissue (DCE-based score: 5 for both readers). The tumor shows homogeneous SI in T2-WI, DWI, ADC, and DCE without any signs of severe necrosis. The final VIRADS scores for both readers were 5. **(e)** Pathological specimens obtained via radical cystectomy confirm muscle-invasive PUC histology. High-power H&E staining highlights there is no indication of necrosis in the histologic examination

**Fig. 5:**
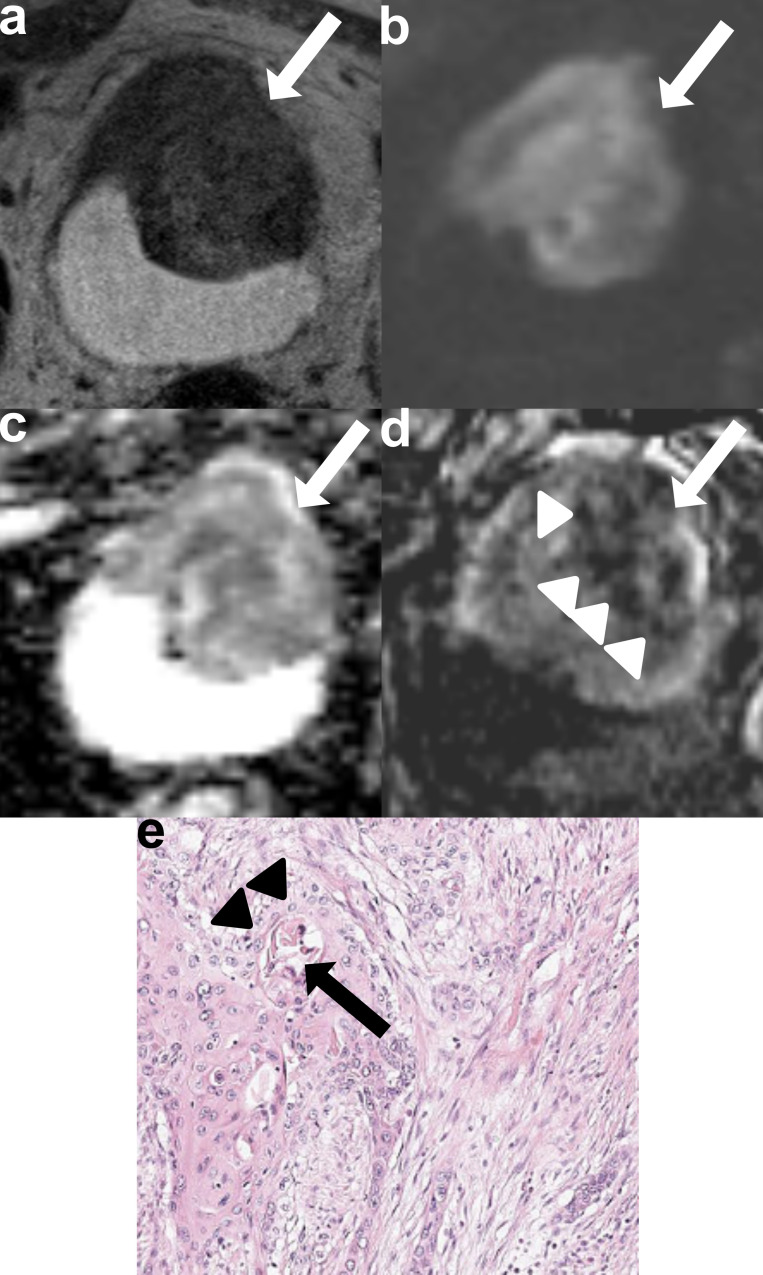
Representative mpMRI and histological images of a correctly diagnosed MIBC (pT2) with VUC histology (squamous differentiation) in a 68-year-old female patient. **a**. Axial T2-WI sequence shows a broad-based, papillary tumor on the anterior bladder wall with low and heterogeneous SI. The tumor interrupts the low SI line of the muscular layer and extends into the perivesical fatty tissue (T2-WI-based score: 5 for both readers) (arrow). **(b)** DWI sequence clearly demonstrates a tumor with high and heterogeneous SI that disrupts the low SI line of the muscular layer and extends into the perivesical fatty tissue (DWI-based score: 5 for both readers) (arrow). **(c)** ADC maps reveal the tumor’s low and heterogeneous SI (arrow). **(d)** DCE sequence shows heterogeneous and early enhancement of the tumor, with disruption of the muscular layer’s low SI, indicating tumor infiltration (arrow). The tumor also extends into the perivesical fatty tissue (DCE-based score: 5 for both readers). Areas of intratumoral low SI suggest necrosis (arrowheads). The final VIRADS scores for both readers were 5. **(e)** Pathological specimens obtained via radical cystectomy confirm muscle-invasive VUC histology with squamous differentiation. High-power H&E staining highlights keratinization (arrow) and intercellular bridges (arrowheads)

The diagnostic performance of all sequences and final VI-RADS scores for detecting MIBC in the PUC and VUC cohorts is summarised in Table 5. In the PUC cohort, AUC values for T2-WI, DWI, DCE, and final VI-RADS scores were 0.944/0.917, 0.972/0.944, 0.972/0.972, and 0.972/0.972, respectively, for readers 1 and 2 (Fig. 2). In the VUC cohort, the corresponding AUC values were lower, at 0.811/0.742, 0.838/0.743, 0.838/0.781, and 0.838/0.781, respectively (Fig. 3). Although diagnostic performance was better in the PUC cohort, there were no significant differences in sensitivity, specificity, accuracy, or AUC values between the cohorts for all sequence and final VI-RADS scores (*p* > 0.05). The Cochran-Armitage test showed a positive correlation between higher VI-RADS scores and increased MIBC rates (*p* < 0.001). Representative mpMRI findings of BCs with PUC and VUC histology are shown in Figs. 4 and 5.

**Table 5:** Diagnostic performance of sequences-based and final VI-RADS scores for detecting MIBC in both cohorts

	PUC	VUC	*p* value
**Reader 1**			
T2-WI sensitivity (%)	88.8	67.7	0.06
T2-WI specificity (%)	100	91.6	0.61
T2-WI accuracy (%)	88	87.2	0.91
DWI sensitivity (%)	94.4	84.2	0.46
DWI specificity (%)	100	94.6	0.72
DWI accuracy (%)	96	92.2	0.73
DCE sensitivity (%)	94.4	84.7	0.41
DCE specificity (%)	100	94.6	0.71
DCE accuracy (%)	96	90.6	0.65
VI-RADS sensitivity (%)	94.4	86.8	0.63
VI-RADS specificity (%)	100	94.6	0.71
VI-RADS accuracy (%)	96	90.6	0.61
**Reader 2**			
T2-WI sensitivity (%)	83.3	64.7	0.08
T2-WI specificity (%)	100	94.6	0.72
T2-WI accuracy (%)	92	89.2	0.85
DWI sensitivity (%)	88.8	78.9	0.43
DWI specificity (%)	100	91.8	0.71
DWI accuracy (%)	92	90.1	0.88
DCE-MRI sensitivity (%)	94.4	82.8	0.32
DCE-MRI specificity (%)	100	89.3	0.41
DCE-MRI accuracy (%)	96	88.2	0.57
VI-RADS sensitivity (%)	94.4	83.2	0.45
VI-RADS specificity (%)	100	94	0.69
VI-RADS accuracy (%)	96	88.2	0.58

### Inter-reader agreement of VI-RADS scores

Inter-reader agreement was excellent for T2-WI-based, DWI-based, DCE-based, and final VI-RADS scores, with κ values of 0.881, 0.903, 0.885, and 0.685 in the PUC cohort, and 0.840, 0.801, 0.862, and 0.853 in the VUC cohort, respectively.

## Discussion

Our study confirmed that VI-RADS is a reliable tool for evaluating muscle invasion in both VUC and PUC cases, with high accuracy rates and strong inter-reader agreement regardless of experience level. Our results are consistent with those of Arita et al., who reported that VI-RADS scoring achieves high diagnostic accuracy in VUC cases [15]. Additionally, our study highlighted that visual MRI findings (heterogeneous SI on T2-WI, DCE, and the presence of necrosis) and quantitative measures (ADC_n_ values) exhibit high accuracy in differentiating PUC from VUC and predicting VUC cases. Our results align with the promising outcomes reported by Park et al. regarding the use of visual MRI findings for PUC-VUC discrimination and demonstrate that both visual and quantitative MRI parameters are effective in distinguishing PUC from VUC [17].

When examining the sequences separately for predicting MIBC in the VUC cohort, we observed that the T2-WI-based VI-RADS score the lowest sensitivity (VUC: 67.7–64.2%; PUC: 88.8–83.3%), specificity (VUC: 94.6–94.6%; PUC: 100–100%), accuracy (both cohorts: 89.2–87.2%), and AUC (VUC: 0.811–0.742; PUC: 0.944–0.917) for predicting MIBC in both cohorts for both readers. While the PUC cohort outperformed VUC, differences were not statistically significant (*p* > 0.05, for both readers). These findings are consistent with existing literature [11, 12, 15, 16, 18]. This outcome is expected, as the T2-WI sequence in VI-RADS scoring primarily assesses tumor morphology rather than muscle invasion. Therefore, we do not recommend relying solely on the T2-WI-based VI-RADS score for predicting muscle invasion in either PUC or VUC cases.

The DWI-based VI-RADS score demonstrated higher sensitivity, specificity, accuracy, and AUC for predicting muscle invasion in both cohorts, with better performance by the more experienced reader (VUC: 84.2%, 94.6%, 92.2%, and 0.838; PUC: 94.4%, 100%, 96%, and 0.972) compared to the less experienced reader (VUC: 78.9%, 91.8%, 90.1%, and 0.743; PUC: 88.8%, 100%, 92%, and 0.944). Despite slightly lower performance in the VUC cohort, differences were not statistically significant (*p* > 0.05, for both readers). These results affirm that DWI is a key sequence for assessing muscle invasion in BC, consistent with existing literature [12, 19, 20]. However, several challenges can affect the diagnostic accuracy of DWI, particularly in VUC cases. VUC cases often exhibit heterogeneous and low-to-medium SI on DWI due to low cell density, microinvasion into the muscle layer, widespread necrosis, and consequently a lower predictive value for assessing muscle invasion. In our study, as in the literature, lower accuracy was observed in predicting muscle invasion in VUC cases, especially with less experienced readers [15, 21]. While DWI is critical for evaluating muscle invasion in PUC cases, we caution that relying solely on DWI in VUC cases may yield misleading results. We recommend interpreting DWI findings in conjunction with DCE sequences for more accurate predictions.

The DCE-based VI-RADS score demonstrated high diagnostic performance for muscle invasion assessment in both cohorts, with sensitivity (82.8–94.4%), specificity (89.3–100%), accuracy (88.2–96%), and AUC (0.781–0.972) for both readers. While the PUC cohort showed marginally higher accuracy (96% vs. 90.6% for the more experienced reader) and sensitivity compared to VUC, differences were not statistically significant (*p* > 0.05, for both readers). Arita et al. recently reported the superior diagnostic performance of mpMRI over bpMRI for VUC, largely due to the DCE-based VI-RADS score—a finding supported by our results [16]. We believe that the high diagnostic performance of the DCE-based VI-RADS score, in the VUC cohort, is due to the disruption of the tumor’s inner layer caused by the heterogeneous cell density and aggressive nature of VUC cases, as well as the early detection of contrast enhancement in the muscularis propria. Therefore, although the DCE-based VI-RADS score has been shown to provide a higher estimate of tumor stage in PUCs, we consider it an indispensable tool for accurately assessing muscle invasion in VUCs.

The final VI-RADS score demonstrated high diagnostic performance for predicting muscle invasion, with superior sensitivity (83.2–94.4%), specificity (94–100%), accuracy (88.2–96%), and AUC (0.781–0.972) in the PUC cohort compared to VUC for both readers. However, differences between cohorts were not statistically significant (*p* > 0.05). MIBC rates correlated strongly with higher VI-RADS scores across all sequences (T2-WI, DWI, DCE) in both cohorts (*p* < 0.001). Notably, the final VI-RADS score was significantly higher in the VUC cohort (*p* < 0.001). There is only one study in the literature comparing the performance of VI-RADS in predicting muscle invasion in PUC and VUC cohorts. Arita et al. reported findings similar to ours, with AUCs for MIBC prediction in the PUC and VUC cohorts at 0.93–0.94 and 0.89–0.92, respectively, without significant differences (*p* > 0.05) [15]. Although the AUC values we found in the VUC cohort were lower than those in Arita et al.’s study, our results were similar. The difference may be due to the smaller number of VUC cases in our study and the varying experience levels of the readers. The higher final VI-RADS score in the VUC cohort compared to the PUC cohort may be attributed to the tendency of VUC cases to be more invasive. In our study, both muscle invasion and tumor grade were higher in the VUC cohort. Our study confirms that VI-RADS is a reliable tool for evaluating muscle invasion in both PUC and VUC cases.

In our study, we found that the VUC cohort had a significantly larger tumor diameter compared to the PUC cohort (*p* < 0.001). The long-to-short axis ratio of tumors was also relatively higher in the VUC cohort than in the PUC cohort, but the difference was not statistically significant (*p* > 0.05 for both readers, respectively). There were no significant differences in tumor morphology between the cohorts (*p* = 0.546). These findings align with previously reported results in the literature [15, 23]. Given that VUCs are known to grow laterally and exhibit more diffuse patterns, it is unsurprising that VUC cases demonstrated a larger tumor diameter than PUC cases, as confirmed in our study. Huang et al. reported that tumor length, long-to-short axis ratio, and morphology contributed to radiomics models, albeit with low significance in distinguishing VUCs from PUCs [22]. However, based on the findings, we believe that these features, apart from largest tumor diameter, are not reliable markers for distinguishing VUCs from PUCs.

In our study, heterogeneous SI on T2-WI, DCE patterns, the presence of necrosis, and lower ADCn values were significantly more common in the VUC cohort (all *p* < 0.05 for both readers, except DCE for reader 2: *p* = 0.01), effectively differentiating VUC from PUC. Three MRI features (heterogeneous SI on T2-WI, DCE, and presence of necrosis) were identified as independent predictors of VUC. Park et al. reported similar findings in their study [17]. The higher prevalance of necrosis in VUC reflects its aggressive nature compared to PUC and may overlap with heterogeneous SI and lower ADC_n_ values. VUCs have poor responses to cisplatin-based chemotherapy, but their high PD-L1 expression makes them candidates for immunotherapy [7, 23, 24]. However, pathological discrepancies between TUR-B and RC complicate VUC diagnosis. Therefore, distinguishing PUC from VUC using simplified MRI features without the need for pathological confirmation is crucial to determining treatment strategies and improving patient outcomes. Our results demonstrate that both visual and quantitative MRI features can effectively distinguish PUC from VUC.

Our study demonstrated excellent inter-reader agreement in predicting MIBC across all sequences (T2-WI, DWI and DCE) and the final VI-RADS score for both cohorts. Arita et al. reported good to excellent inter-reader agreement among four readers [15]. Inter-reader agreement was also good for identifying heterogeneous SI on T2-WI, DCE, and tumor necrosis—key radiological independent predictors of VUC. Our findings suggest that VI-RADS can be applied with high accuracy across readers with varying levels of experience in both PUC and VUC cohorts. We believe that the visual MRI features considered predictive for the VUC defined in our study can also be utilized with high accuracy.

Our study has several limitations. First, its retrospective design, which relies on reinterpretation by two readers, may introduce bias. Second, TUR-B served as the reference standard in 68.2% of the patients, which is a limitation because the likelihood of identifying MIBC increases when RC is performed. To address potential discrepancies between TUR-B and RC results, a second TUR-B was performed in 90% of high-risk NMIBC cases to remove residual tumor tissue and confirm the disease stage. Third, 31.2% of VUC cases received NAC prior to RC. Consequently, TUR-B could not accurately determine the proportion of variant histology in resected specimens, and RC could not fully reflect the histological composition of the tumors. As a result, the impact of differential VUC characteristics on diagnostic performance should be evaluated in larger cohorts of patients undergoing RC without NAC. Fourth, ADC measurements are highly susceptible to errors. However, we sought to mitigate these inaccuracies by employing a reference ADC, evaluating tumors through multiple parameters, and utilizing two independent readers. Finally, the number of VUC cases in our study is relatively small. However, unlike other studies, which typically focus on the index lesion in cases with multiple tumors, we evaluated each tumor individually, providing more comprehensive and realistic results. Nevertheless, our findings need to be confirmed in larger patient groups.

## Conclusion

In light of the growing need for non-invasive imaging techniques to detect VUCs, our study demonstrates that the VI-RADS can be applied with high diagnostic accuracy in both PUC and VUC, irrespective of reader experience. Furthermore, specific radiological features—including the presence of necrosis on preoperative MRI, heterogeneous SI on T2-WI, and DCE images—were identified as independent predictors of VUC. These findings hold significant potential to inform treatment strategies and improve patient management, underscoring the clinical utility of mpMRI in guiding therapeutic decisions and optimizing outcomes.

## Data Availability

No datasets were generated or analysed during the current study.
